# Pulmonary involvement in Gaucher disease patients receiving enzyme replacement therapy: a cross-sectional analysis of radiological and functional lung abnormalities

**DOI:** 10.1007/s00431-026-07476-3

**Published:** 2026-11-10

**Authors:** Sara A. Bahgat, Amal Osman, Amira Hussein, Ashraf M. Abdelrahman, Sherine M. Elzeiny, Tarek Eldesoky, Ahmed Megahed

**Affiliations:** 1 Department of Pediatrics, Faculty of Medicine, Mansoura University, Mansoura, Egypt; 2 Pediatric Allergy and Immunology Unit, Department of Pediatrics, Faculty of Medicine, Mansoura University, Mansoura, Egypt; 3 Pediatric Cardiology Unit, Department of Pediatrics, Faculty of Medicine, Mansoura University, Mansoura, Egypt; 4 Mansoura University Children’s Hospital, Mansoura University, Mansoura, Egypt; 5 Pediatric Gastroenterology and Hepatology Unit, Department of Pediatrics, Faculty of Medicine, Mansoura University, Mansoura, Egypt

**Keywords:** Gaucher disease, Pulmonary manifestations, Interstitial lung disease, HRCT chest, Pulmonary function tests

## Abstract

Pulmonary involvement in Gaucher disease (GD) is underrecognized and may range from asymptomatic radiological abnormalities to respiratory impairment. This study assessed pulmonary involvement and its associations with disease phenotype, genotype, and pulmonary function in patients receiving long-term enzyme replacement therapy (ERT). In this single-center cross-sectional study, 52 genetically confirmed patients with GD receiving ERT underwent clinical assessment and high-resolution computed tomography (HRCT). Spirometry was performed in 34 cooperative patients aged ≥ 6 years. Clinical characteristics, genotype, baseline glucosylsphingosine (lyso-GL1), and disease phenotype were analyzed in relation to pulmonary findings. Respiratory symptoms were present in 19/52 (36.5%) patients. HRCT demonstrated interstitial abnormalities suggestive of interstitial lung disease (ILD) in 24/52 (46.2%), predominantly ground-glass opacities and interstitial thickening. These abnormalities were more frequent in type 3 than type 1 GD (*P* = 0.001). Homozygous L483P was associated with HRCT abnormalities suggestive of ILD in univariable analysis (*P* = 0.013). Among 34 patients undergoing spirometry, 11 (32.4%) had abnormal results, including obstructive (8.8%), restrictive (11.8%), and mixed (11.8%) patterns. Abnormal spirometry was associated with HRCT abnormalities (*P* < 0.001), while three patients had radiological abnormalities despite preserved spirometry. On multivariable analysis, higher baseline lyso-GL1, type 3 phenotype, and kyphoscoliosis were independently associated with HRCT abnormalities suggestive of ILD.

*Conclusion*: Pulmonary abnormalities remained common despite long-term ERT, particularly in type 3 GD. HRCT detected interstitial abnormalities in some patients with preserved spirometry, suggesting that structural pulmonary involvement may precede functional impairment. Individualized respiratory surveillance should be considered, particularly in patients with type 3 GD.

**Table:** 

**What is Known**: • *Pulmonary involvement in Gaucher disease ranges from subclinical radiological abnormalities to respiratory impairment and is more frequent in type 3 disease. Its relationship with genotype and lung function remains incompletely defined*.	
**What is New**: • *Pulmonary abnormalities were frequent despite long-term enzyme replacement therapy, particularly in type 3 disease. HRCT detected interstitial abnormalities in patients with normal spirometry, while type 3 phenotype, higher baseline lyso-GL1, and kyphoscoliosis independently predicted pulmonary involvement*.

**What is Known**:

• *Pulmonary involvement in Gaucher disease ranges from subclinical radiological abnormalities to respiratory impairment and is more frequent in type 3 disease. Its relationship with genotype and lung function remains incompletely defined*.

**What is New**:

• *Pulmonary abnormalities were frequent despite long-term enzyme replacement therapy, particularly in type 3 disease. HRCT detected interstitial abnormalities in patients with normal spirometry, while type 3 phenotype, higher baseline lyso-GL1, and kyphoscoliosis independently predicted pulmonary involvement*.

## Introduction

Gaucher disease (GD) is a rare autosomal recessive lysosomal storage disorder caused by pathogenic variants in the Glucocerebrosidase 1 (GBA1) gene, encoding glucocerebrosidase [1]. Enzyme deficiency leads to accumulation of glucosylceramide within macrophages, resulting in multisystem involvement affecting the liver, spleen, bone marrow, skeleton, lungs, and, in neuronopathic forms, the central nervous system [2, 3].

Although pulmonary involvement has long been considered infrequent in GD, increasing evidence suggests that respiratory manifestations are more common than previously recognized. Pulmonary abnormalities result mainly from Gaucher cell infiltration of the lungs and pulmonary vessels, causing various structural and functional respiratory abnormalities [4]. Chest CT may reveal interlobular septal thickening, ground-glass opacities, interstitial infiltration, and vascular abnormalities, while fibrosis, nodules, and lymphadenopathy are less frequent. This heterogeneity complicates the recognition and standardized assessment of pulmonary involvement [4, 5].

Pulmonary function testing may detect physiological abnormalities before clinically apparent respiratory disease [6]. However, published data regarding spirometric abnormalities in GD and their relationship with radiological findings remain limited.

Genotype may affect pulmonary involvement; homozygous L483P (formerly L444P) is more common in intrinsic pulmonary disease, but genotype–phenotype relationships remain unclear [4, 5, 7].

Enzyme replacement therapy (ERT) has significantly improved outcomes in GD; however, its effect on pulmonary disease is variable [8–10], and progressive or persistent respiratory abnormalities have been reported despite prolonged ERT [11, 12]. Few studies have assessed pulmonary imaging, function, and genotype together in GD, especially in Middle Eastern and North African populations. This study aimed to characterize pulmonary manifestations in Egyptian GD patients and evaluate their relationships with HRCT, spirometry, GBA1 genotype, and clinical phenotype.

## Patients and methods

This single-center cross-sectional observational study combined retrospective extraction of clinical data from medical records with prospective assessment of pulmonary involvement during a single study visit. The study included all consecutive patients with a confirmed diagnosis of GD who were under regular follow-up and receiving ERT at the Gastroenterology Unit, Mansoura University Children’s Hospital between July 2023 and July 2025. Owing to the rarity of GD, all eligible patients were included without formal sample size calculation. The cohort comprised both pediatric and adult patients, as our center provides lifelong follow-up for patients diagnosed with Gaucher disease during childhood.

Patients were diagnosed based on deficient peripheral blood glucocerebrosidase activity and identification of pathogenic GBA1 variants, in accordance with established diagnostic guidelines [13].

Patients without primary neuronopathic involvement were classified as type 1, whereas those with a chronic neurological phenotype compatible with neuronopathic GD were classified as type 3 based on longitudinal clinical assessment [14–18]. Horizontal supranuclear gaze abnormalities were specifically assessed in accordance with the 2020 consensus definition [14]. Other neurological manifestations, including strabismus, seizures, and dysphagia were recorded as associated neurological findings [15–17]. The neurological phenotype was considered in the context of the longitudinal clinical course, given the variable clinical spectrum of neuronopathic GD [14, 17]. No alternative neurological diagnosis was identified during follow-up.

Clinical data; disease phenotype; baseline laboratory results (hemoglobin, platelet counts, chitotriosidase activity, and lyso-GL1 levels); treatment history; and genotype information were extracted from medical records. Available echocardiographic assessments were reviewed by the pediatric cardiologist for evidence of pulmonary hypertension according to clinical and echocardiographic criteria [19–21].

### Anthropometric, biomarker assessment, and liver and spleen volumetry

Weight, height, and BMI were measured and interpreted using WHO standards for children < 2 years, 2000 CDC Growth Charts for ages 2–20 years, and WHO criteria for adults [22–24]. Chitotriosidase and lyso-GL1 were assessed using standardized or assay-specific reference ranges [13, 25]. Liver and spleen volumes were measured by HRCT volumetry using age- and body-size-adjusted references [26–29].

During the study visit, patients underwent clinical evaluation and disease severity assessment using validated scoring systems. The Pediatric Gaucher Severity Score (*PGS3*) was used in children (mild ≤ 6, moderate 6–8, severe ≥ 9), the Gaucher Disease Severity Scoring system(GD1-DS3)  in adults with type 1 GD (mild (<3) , moderate (3 to <6) , marked/severe (≥6 ) , and a *modified neurological severity* score in neuronopathic disease [30–32]. Eligible patients underwent spirometry and HRCT.

### Spirometry

Pulmonary function testing (PFT) was performed using a computerized spirometer *(MasterScreen Pediatrics, Jaeger, Germany)* in accordance with the American Thoracic Society/European Respiratory Society (ATS/ERS) standards [33]. Spirometry was performed in 34 cooperative patients aged ≥ 6 years. The remaining 18 patients were not assessed because they were either younger than 6 years or unable to perform acceptable spirometric maneuvers because of limited cooperation or neurological impairment. At least three acceptable maneuvers were obtained, and the highest values were recorded. Spirometric values were expressed as percentages of the Global Lung Function Initiative (GLI)−2012 predicted values adjusted for age, sex, and height. Measured parameters included forced vital capacity (FVC), forced expiratory volume in one second (FEV₁), FEV₁/FVC ratio, and forced expiratory flow between 25 and 75% of vital capacity (FEF₂₅–₇₅) [33].

Spirometry was classified as *normal* (FVC and FEV₁ ≥ 80% predicted with a preserved FEV₁/FVC ratio), *obstructive* (reduced FEV₁ and FEV₁/FVC ratio), *restrictive* (reduced FVC with a normal or increased FEV₁/FVC ratio), or *mixed* (concurrent reductions in FEV₁, FVC, and FEV₁/FVC ratio) according to standard criteria. Because total lung capacity was not measured, restrictive spirometric abnormalities were considered suggestive rather than diagnostic of restrictive lung disease [33, 34].

### Radiology

HRCT was performed using *a Revolution EVO 128-slice CT scanner (GE Healthcare, USA)* with dose-adjusted radiation exposure. Images were reviewed by a consultant radiologist blinded to clinical data. Pulmonary involvement was defined as HRCT evidence of interstitial abnormalities suggestive of interstitial lung disease (ILD), including ground-glass opacities, interstitial thickening, and reticulation. These findings were considered radiological evidence of interstitial pulmonary involvement rather than a definitive clinical diagnosis of ILD. Other findings, including nodules and lymphadenopathy, were recorded. As no GD-specific CT scoring system exists, the Warrick score was used as an exploratory semi-quantitative measure, classifying interstitial lung involvement as mild (0–7), moderate (8_15), or severe (> 15) [35, 36].

### Statistical analysis

Statistical analysis was performed using IBM SPSS Statistics version 26 (SPSS Inc., PASW Statistics for Windows version 26. Chicago: SPSS Inc.). Continuous variables were presented as mean ± SD or median (range), and categorical variables as frequencies and percentages. Group comparisons used appropriate parametric or non-parametric tests. Variables selected based on statistical significance and clinical relevance were entered simultaneously into a multivariable binary logistic regression model using the Enter method. Adjusted odds ratios (ORs) with 95% confidence intervals (CIs) were reported, and *P* < 0.05 was considered statistically significant. Potential collinearity among phenotype, L483P genotype, and kyphoscoliosis was assessed using cross-tabulations and variance inflation factors (VIFs).

## Results

Fifty-two patients with GD included the following: 33 (63.5%) males and 19 (36.5%) females; 13 (25%) were adults. Type 1 GD was diagnosed phenotypically in 33 (63.5%) patients and type 3 GD in 19 (36.5%). All patients were receiving ERT for a median of 11.5 years, with imiglucerase administered to fifty patients and velaglucerase alfa to two patients. Patients with type 3 GD received significantly higher ERT doses than those with type 1 GD (*P* < 0.05). Baseline characteristics are summarized in Table 1.

**Table 1:** Demographic, clinical, and laboratory characteristics of Gaucher disease patients

	Gaucher disease patients (*n* = 52)	Reference/normal values
**Age** (years)	14.6 (2.5–33.4)	—
**Age of presentation** (years)	0.9 (0.08–20)	—
**Age of diagnosis** (years)	1.3 (0.5–21)	—
**Consanguinity**	39 (75%)	—
**Family history**	28 (53.8%)	—
**Weight** (Kg)	35.5 (11–81)	Age- and sex-specific
**Height** (cm)	143 (85–185)	Age- and sex-specific
**Body mass index(BMI)** (kg/m^2^)	17.6 (13–28)	Age- and sex-specific
**Baseline hemoglobin** (g/dL)	9 (4–13)	Age- and sex-specific [37]
**Baseline platelets** (× 10^9^/L)	112 (34–366)	Age-specific [38, 39]
**Baseline chitotriosidase activity** (µmol/L/h)	0.36 (0–3)	Assay-specific [13]
**Baseline lyso-GL1 (glucosylsphingosine)** (ng/ml)	314.5 (67–1748)	Assay-specific [25]
**Spleen** (cm^3^)	260 (110–570)	Age-/body-size-adjusted [28, 29]
**Liver** (cm^3^)	950 (400–2050)	Age-/body-size-adjusted [26, 27]
**Disease severity according to age and phenotype-specific validated scores** Mild (*n* = 46) Moderate (*n* = 4) Severe (*n* = 2)	46 (88.5%) 4 (7.7%) 2 (3.8%)	—
**Echocardiographic evidence of pulmonary hypertension** (*n* = 13) Mild elevated PASP (*n* = 12) Moderate elevated PASP (*n* = 1)	12 (23.1%) 1 (1.9%)	Mild elevated estimated echocardiographic PASP 30–40 mmHg, Moderate elevated estimated echocardiographic PASP 40–70 mmHg [20, 21]
**Delay in ERT initiation** (years)	0.16 (0–4)	—
**Age at ERT initiation** (years)	2 (0.08–22)	—
**ERT duration** (years)	11.5 (1.5–19.5)	—

Data are presented as median (range) and number (percentage). Reference values were age-, sex-, body-size-, or assay-specific, as applicable. *PASP* estimated pulmonary artery systolic pressure. Disease severity was assessed using the appropriate validated scoring system according to age and phenotype: *PGS3* for children with type 1 GD, *GD1-DS3* for adults with type 1 GD, and the *modified neurological severity score* for patients with type 3 GD

### Genotype distribution

**Fig. 1:**
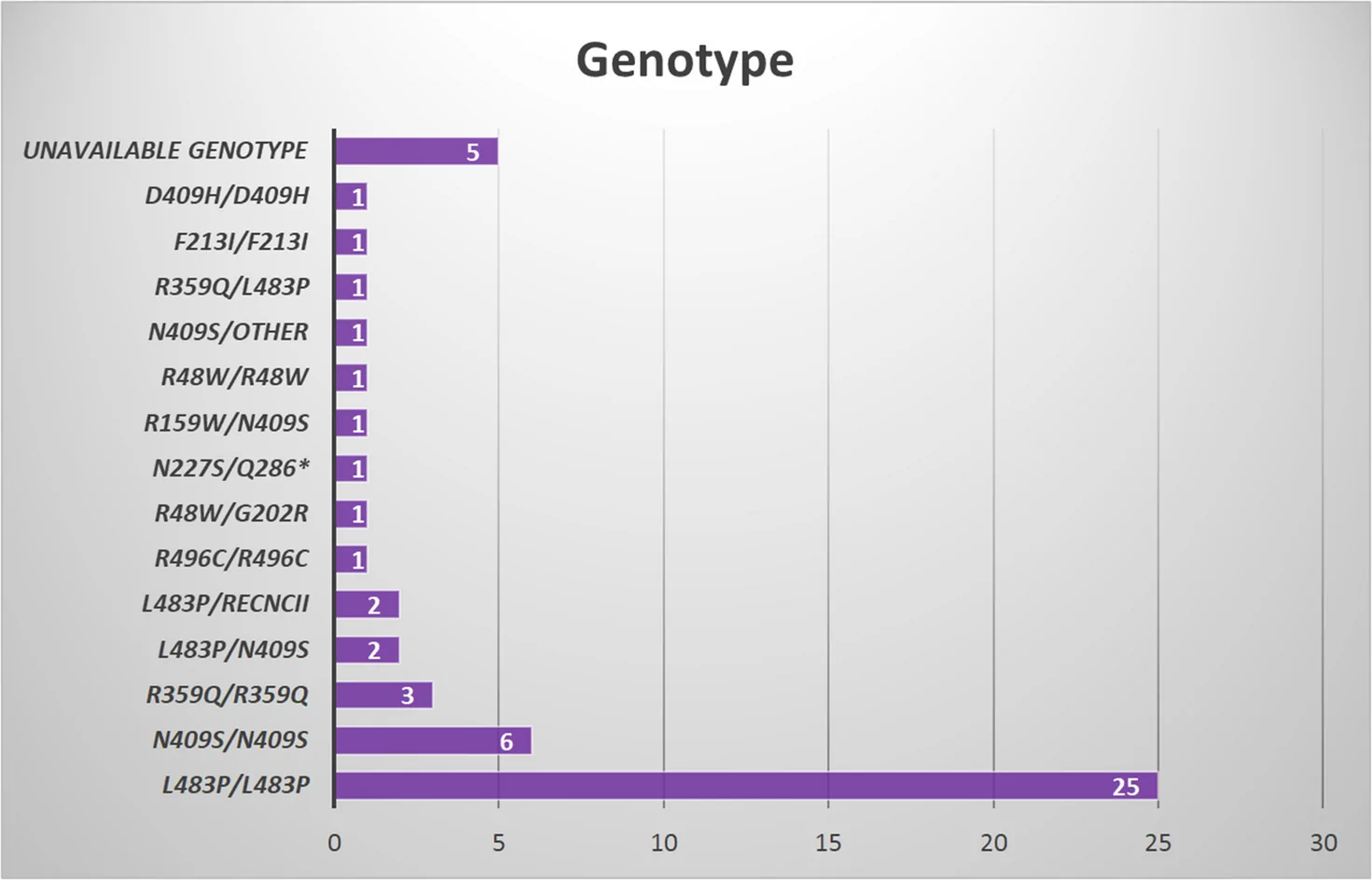
Distribution of GBA1 genotypes among patients with Gaucher disease

The most frequent genotype was *homozygous L483P* (L444P), identified in 25 patients (48.1%), followed by *homozygous N409S* (N370S) in six patients (11.5%) and homozygous R359Q in three patients (5.8%). Compound heterozygous L483P/N409S and L483P/RecNciI variants were detected in two patients each (3.8%). Several rare *GBA1* genotypes were identified, each occurring in a single patient (1.9%), including homozygous and compound heterozygous variants such as (R496C), (R48W/G202R), (N227S/Q286*), (R159W/N409S), (R359Q/L483P), (F213I), and (D409H/D409H), and genotype data were unavailable in five cases (9.6%)) Fig. 1).

Homozygous L483P was present in most patients with type 3 GD (*P* < 0.001), whereas the N409S variant was predominantly observed in patients with type 1 GD***.***

### Clinical findings

The most common initial manifestations were abdominal enlargement (86.5%), anemia (78.8%), and thrombocytopenia (57.7%). Failure to thrive, recurrent chest infections, and developmental delay occurred in 25%, 23%, and 19.2%, respectively; 4/52 (7.7%) were diagnosed by asymptomatic family screening. Type 3 GD showed significantly more neurological and musculoskeletal manifestations: supranuclear gaze palsy and kyphoscoliosis (13/19, 68.4% each); strabismus and dysphagia (8/19, 42.1% each); and seizures (3/19, 15.8%). Respiratory symptoms occurred in 19/52 (36.5%), mainly dyspnea (18/19, 94.7%): New York Heart Association (NYHA), class I (10/18, 55.6%), class II (4/18, 22.2%), class III (4/18, 22.2%); recurrent infections (10/19, 52.6%) and chronic cough (1/19, 5.3%).

### Radiological findings

**Fig. 2:**
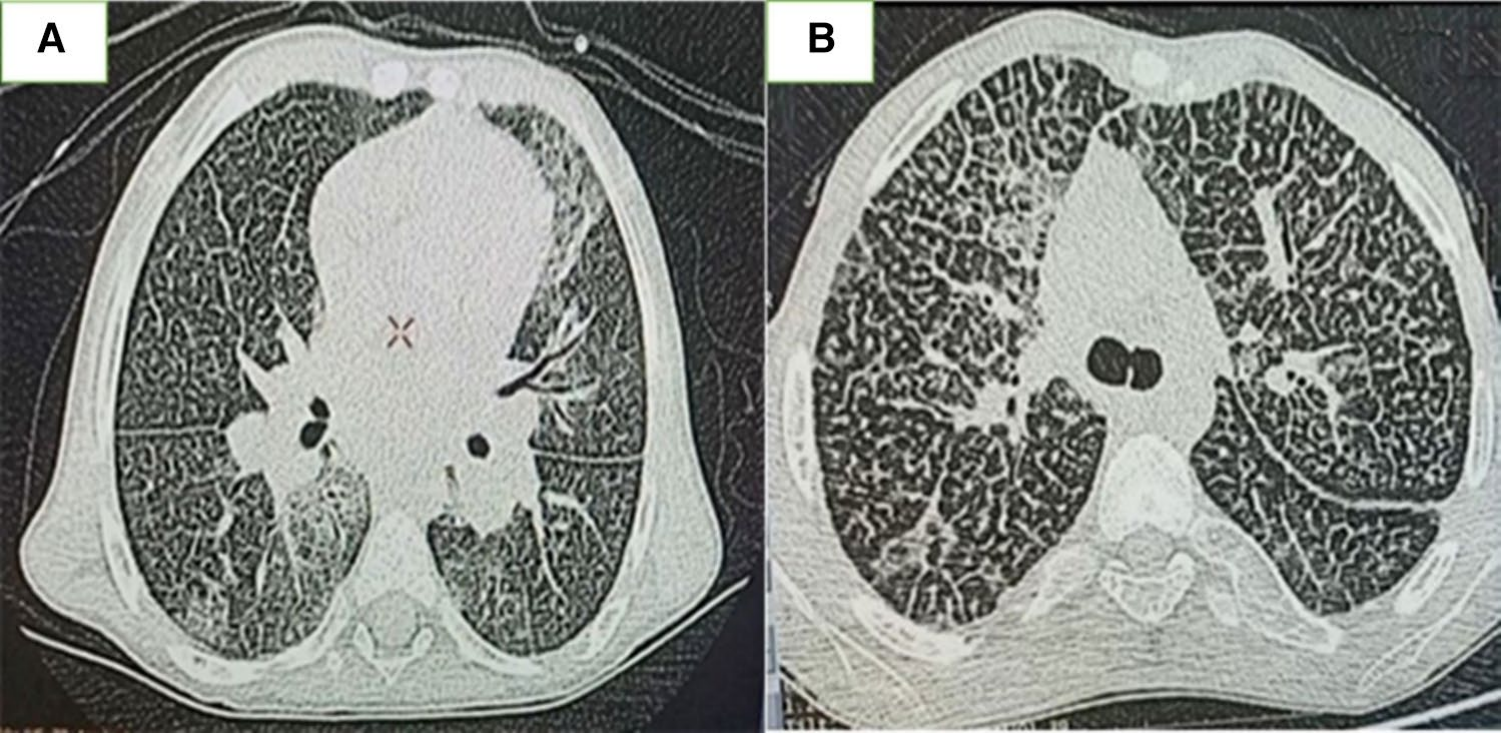
Representative HRCT findings in two patients with Gaucher disease.** A** HRCT of a 6-year-old male patient with chronic cough demonstrating extensive interstitial thickening, bilateral ground-glass opacities, and areas of consolidation. **B** HRCT of a 20-year-old asymptomatic male patient demonstrating diffuse interstitial thickening, widespread ground-glass opacities, and basal consolidation (both patients carried the *homozygous L483P*)

HRCT revealed pulmonary interstitial abnormalities suggestive of ILD in 24 patients (46.2%). Pulmonary involvement was significantly more frequent in type 3 GD compared with type 1 GD (84.2% vs. 24.2%, *P* = 0.001). Ground-glass opacities and interstitial thickening were the most common abnormalities (Table 2) (Fig. 2).Warrick scores ranged from 1 to 9 (median 3); most patients had mild (70.8%) or moderate (29.2%) interstitial involvement, with no severe cases.

**Table 2:** HRCT patterns of interstitial lung abnormalities in patients with Gaucher disease (*n* = 24)

**HRCT-defined interstitial abnormalities**	*n* = 24
Interstitial thickening alone	5 (20.8%)
Ground glass opacities (GGO) alone	5 (20.8%)
Consolidation ± GGO/atelectasis	5 (20.8%)
Interstitial thickening + GGO ± (air trapping/consolidation)	7 (29.2%)
Fibrotic bands	2 (8.3%)
**Other HRCT findings**	***n* = 4**
Small nodules (pulmonary/subpleural)	2 (8.3%)
Mediastinal lymphadenopathy	2 (8.3%)

Data are presented as numbers (percentages)

Patients with HRCT findings suggestive of ILD were younger at presentation and diagnosis and had lower weight, height, and body mass index (BMI), together with higher baseline lyso-GL1 levels (all *P* < 0.05). HRCT-defined interstitial abnormalities were significantly associated with the type 3 phenotype, homozygous L483P genotype, higher disease severity scores, pulmonary manifestations, kyphoscoliosis, and abnormal spirometry (Table 3). Among 34 patients who underwent both HRCT and spirometry, 20/23 with normal HRCT had normal spirometry, whereas 8/11 with ILD-suggestive HRCT had abnormal spirometry. Notably, three patients had HRCT evidence of interstitial abnormalities despite normal spirometry. HRCT abnormalities were significantly associated with abnormal spirometry (*P* < 0.001).

**Table 3:** Characteristics of patients with and without HRCT findings suggestive of interstitial lung disease

	No ILD on HRCT (*n* = 28)	HRCT suggestive of ILD (*n* = 24)	***P*** value
**Age** (years)	16 (2.6–33.4)	11.2 (2.8–23.1)	0.09
**Age of presentation** (years)	1.2 (0.08–20)	0.66 (0.08–2.3)	0.032*
**Age at diagnosis** (years)	1.9 (0.58–21)	1 (0.5–3)	0.01*
**Weight** (kg)	49 (16–81)	27 (11–62)	0.003*
**Height** (cm)	155 (95–185)	128 (85–167)	0.007*
**BMI** (kg/m^2^)	19.98 ± 3.96	17.01 ± 2.64	0.003*
**Age group** Pediatric (*n* = 39) Adult (*n* = 13)	18 (46.2%) 10 (76.9%)	21 (53.8%) 3 (23.1%)	0.054
**Sex** Male (*n* = 33) Female (*n* = 19)	15 (45%) 13 (68%)	18 (55%) 6 (32%)	0.110
**Phenotype** Type 1 (*n* = 33) Type 3 (*n* = 19)	25 (75.8%) 3 (15.8%)	8 (24.2%) 16 (84.2%)	0.001*
**Homozygous L483P** L483P (*n* = 25) Not L483P (*n* = 27)	9 (36%) 19 (70%)	16 (64%) 8 (30%)	0.013*
**Baseline hemoglobin** (g/dL)	9.2 (4–12)	9 (4–13)	0.259
**Baseline platelets** (L/10^9^)	102 (63–233)	141 (34–366)	0.811
**Baseline lyso-GL1** (ng/mL)	229.05 (39–564)	424.2 (107–1748)	0.006*
**Spleen (**cm^3^**)**	270 (130–570)	255 (110–550)	0.891
**Liver (**cm^3^**)**	1020 (540–2050)	715 (400–1600)	0.068
**Severity scoring index** Mild (*n* = 46) Moderate (*n* = 4) Severe (*n* = 2)	27 (58.7%) 1 (25%) 0	19 (41.3%) 3 (75%) 2 (100%)	0.039*
**Age at ERT initiation** (years)	2.2 (0.83–22)	1.2 (0.08–5.8)	0.006*
**ERT duration** (years)	12 (3–19.5)	10.25 (1.5–19)	0.45
**ERT start delay** (years)	0.17 (0.1–4.0)	0.4 (0–1.6)	0.640
**Pulmonary manifestations**	1 (5.3%)	18 (94.7%)	< 0.001*
**Kyphoscoliosis**	4 (21%)	15 (79%)	< 0.001*
**Spirometry results** (*n* = 34) Normal (*n* = 23) Abnormal (*n* = 11)	20 (87%) 3 (27%)	3 (13%) 8 (73%)	0.001*
**Echocardiographic findings suggestive of pulmonary hypertension** No elevated echocardiographic PASP (*n* = 39) Elevated estimated echocardiographic PASP (mmHg) (*N* = 13)	21 (53.8%) 7 (53.8)	18 (46.2%) 6 (46.2%)	1.000

Continuous variables are presented as median (minimum–maximum) or mean ± SD, as appropriate. Categorical variables are presented as *n* (%). Percentages for categorical variables are calculated by row. *Statistically significant. *PASP* estimated pulmonary artery systolic pressure

Multivariable logistic regression identified higher baseline lyso-GL1 levels, type 3 phenotype, and kyphoscoliosis as independently associated with abnormal HRCT findings (Table 4). No evidence of problematic multicollinearity was observed among the included predictors (VIF range, 1.004–1.253).

**Table 4:** Multivariable logistic regression analysis of factors associated with HRCT suggestive of interstitial lung disease (ILD)

	OR	95% CI	*P* value
lyso-GL1 (ng/mL)	1.006	1.001–1.011	0.014*
Type 3 phenotype (vs type 1)	39.913	3.446–462.232	0.003*
Kyphoscoliosis	13.422	1.535–117.355	0.019*

*OR* (95% CI) odds ratio with 95% confidence interval

Reference category for phenotype: type 1 GD. *Statistically significant

To illustrate the spectrum of radiological abnormalities observed in our cohort, representative HRCT images from two patients are presented in Fig. 2

## Spirometry results

Spirometry was performed in 34 cooperative patients aged ≥ 6 years. Normal spirometry was observed in 23 patients (67.6%), whereas abnormalities were detected in 11 (32.4%). Obstructive defects occurred in three patients (8.8%), restrictive patterns in four patients (11.8%), and mixed ventilatory defects in four patients (11.8%). Results of spirometry parameters are shown in the Table 5.

**Table 5:** Spirometry results of Gaucher patients

Parameter	% Predicted
FEV1	81 (25–249)
FVC	70 (30–150)
FEV1/FVC	113.5 (51–191)
FEF 25–75%	70 (31–227)

*FEV1* forced expiratory volume in the first second, *FVC* forced vital capacity, *FEF* forced expiratory flow. Data are presented as median (range) of % predicted values

†Available for 34 patients aged ≥ 6 years who were able to perform acceptable spirometry

Restrictive and mixed ventilatory abnormalities were mainly observed in patients with HRCT-defined interstitial abnormalities suggestive of ILD. Three patients had these abnormalities despite normal spirometry. HRCT abnormalities were independently associated with abnormal pulmonary function (OR 14.5, 95% CI 2.0–105.0, *P* = 0.008).

## Impact of enzyme replacement therapy

Long-term ERT significantly improved hemoglobin (median 9.0 to 11.9 g/dL) and platelet counts (median 112 to 195.5 × 10^9^/L; both *P* < 0.001), as well as liver volume (*P* = 0.007) and growth parameters (*P* < 0.001). The median age at ERT initiation was 2.0 years (0.08–22), with a median treatment duration of 11.5 years (1.5–19.5). Patients with HRCT findings suggestive of ILD had a younger age at ERT initiation (*P* = 0.006), while ERT duration and treatment delay were not associated with HRCT suggestive of ILD (*P* > 0.05). Respiratory manifestations, including recurrent chest infections and aspiration episodes, showed limited improvement despite long-term ERT.

## Discussion

The clinical characteristics of our cohort highlight the distinctive epidemiology of Gaucher disease (GD) in Egypt while remaining consistent with international reports. Although type 1 GD was the predominant phenotype (63.5%), type 3 GD accounted for more than one-third of patients, in contrast to Western populations, where type 1 GD represents over 90% of cases, but aligning with previous Egyptian studies reporting a higher frequency of type 3 GD [5, 40, 41]. This distinct distribution may reflect the high prevalence of parental consanguinity and the predominance of the homozygous *L483P* pathogenic variant in the Egyptian population, which is strongly associated with the chronic neuronopathic phenotype [18, 41]. Hepatosplenomegaly, anemia, and thrombocytopenia were the most frequent presenting manifestations, reflecting the characteristic visceral and hematological involvement reported in both Egyptian and international cohorts [18, 40].

The genotypic profile of our patients was also consistent with previous Egyptian studies, with homozygous *L483P* being the predominant pathogenic variant, followed by homozygous *N409S* [5, 18, 42, 43]. *L483P* was identified in most patients with type 3 GD, whereas N409S, either homozygous or compound heterozygous, was predominantly associated with type 1 GD, supporting the established genotype–phenotype correlation reported in Egyptian and international cohorts [44, 45].

Respiratory symptoms occurred in over one-third of our cohort, with nearly two-thirds of symptomatic patients having type 3 GD. Dyspnea was the predominant respiratory manifestation, followed by recurrent respiratory infections. This prevalence is comparable to that reported by Tantawy et al. (33.3% in Egyptian patients) [5]. The predominance of respiratory symptoms in type 3 GD is consistent with previous reports and may reflect the greater systemic disease burden associated with neuronopathic phenotypes [11].

Pulmonary abnormalities suggestive of ILD were detected by HRCT in nearly half of our cohort (46.2%), with ground-glass opacities and interstitial thickening representing the predominant radiological findings. Similar imaging patterns have been consistently described in previous studies of GD, where ground-glass opacities and interstitial changes constitute the most frequent HRCT abnormalities [46, 47]. Although the prevalence of HRCT abnormalities in our cohort was lower than the 64.6% reported by Tantawy et al. in an Egyptian pediatric cohort, it remains considerably higher than that reported in earlier studies relying on chest radiography or clinically overt lung disease, highlighting the superior sensitivity of HRCT for detecting early or subclinical pulmonary involvement [5, 48, 49]. Furthermore, HRCT abnormalities were significantly more frequent in patients with type 3 GD and were associated with the homozygous *L483P* genotype, consistent with previous reports demonstrating the greater susceptibility of neuronopathic GD to pulmonary involvement [4, 5, 7, 9].

In the present study, patients with HRCT abnormalities suggestive of ILD were younger at presentation and diagnosis and had poorer growth, higher baseline lyso-GL1 levels, higher disease severity scores, kyphoscoliosis, and more frequent pulmonary manifestations. Multivariable analysis identified baseline lyso-GL1, type 3 phenotype, and kyphoscoliosis as independently associated with HRCT abnormalities. However, the wide confidence intervals, particularly for type 3 phenotype and kyphoscoliosis, indicate considerable uncertainty in the magnitude of these associations. These findings should therefore be interpreted cautiously and validated in larger multicenter cohorts. Consistent with our findings, Tantawy et al. reported thrombocytopenia, anemia, and higher disease severity scores as predictors of abnormal HRCT findings [5].

A notable finding of the present study was the detection of elevated baseline lyso-GL1 levels as an independent predictor of HRCT abnormalities. lyso-GL1, a direct metabolite of Gb1, is a sensitive biomarker of GD burden and correlates with disease severity and treatment response [50]. Accumulation of Gb1 within lysosomes promotes macrophage activation and inflammatory signaling, which may contribute to tissue injury and disease progression [51, 52]. These mechanisms provide a plausible biological basis for the association between elevated baseline lyso-GL1 levels and pulmonary involvement observed in our cohort.

Elevated PASP on echocardiography was observed in approximately one-quarter of our cohort. Pulmonary hypertension is a recognized complication of GD and may result from Gaucher cell infiltration, interstitial lung disease, and pulmonary vascular remodeling [11, 52]. Its relatively high frequency in our cohort may be related to the predominance of type 3 GD and interstitial lung involvement. However, as assessment was based on echocardiography rather than right heart catheterization, these findings should be considered suggestive rather than diagnostic.

Approximately one-third of the patients who underwent spirometry in the present study demonstrated ventilatory abnormalities, with restrictive and mixed ventilatory defects accounting for most abnormal patterns. These findings are consistent with previous studies showing that pulmonary function abnormalities are common in GD, although the reported prevalence varies according to the pulmonary function parameters assessed and the characteristics of the study population [48, 49]. Kerem et al. reported a higher prevalence of pulmonary function abnormalities (68%) in patients with type 1 GD; however, their assessment included lung volumes and diffusing capacity for carbon monoxide (DLCO) and transfer coefficient for carbon monoxide (KCO), which are more sensitive than spirometry alone for detecting early pulmonary involvement [48]. In contrast, our study evaluated spirometric indices only, which may explain the lower prevalence of ventilatory impairment.

Restrictive and mixed ventilatory defects predominated in our cohort and were observed almost exclusively in patients with HRCT-defined interstitial pulmonary abnormalities suggestive of ILD. This is consistent with Miller et al., who demonstrated that patients with clinically evident pulmonary involvement consistently exhibited restrictive ventilatory impairment with reduced diffusing capacity, whereas patients without radiological lung disease generally had preserved pulmonary function [49]. Notably, three patients had HRCT evidence of ILD despite normal spirometry, suggesting that structural lung involvement may precede functional impairment. These findings highlight the complementary role of HRCT in detecting early pulmonary involvement that may not be identified by spirometry alone.

All patients in our cohort received long-term ERT, with a median treatment duration of 11.5 years. ERT improved hematological and visceral manifestations, including anemia, thrombocytopenia, and hepatomegaly, consistent with previous reports [53–55]. However, pulmonary abnormalities persisted in some patients despite prolonged therapy. The younger age at ERT initiation among patients with HRCT findings suggestive of ILD may reflect earlier diagnosis and treatment of more severe phenotypes, particularly type 3 GD, rather than an adverse effect of early ERT. Persistent pulmonary abnormalities may indicate established structural lung damage, highlighting the importance of early diagnosis and ongoing respiratory surveillance to minimize irreversible pulmonary complications [56–58].

In conclusion, pulmonary abnormalities were common in GD, especially type 3, despite long-term ERT. HRCT detected interstitial abnormalities more frequently than spirometry and identified pulmonary involvement in some patients with preserved spirometry; therefore, early recognition and regular respiratory surveillance are important, particularly in high-risk patients. Combining clinical assessment, pulmonary function tests, and HRCT when indicated may improve detection of pulmonary complications.

## Strengths and limitations

A key strength of this study is the integrated evaluation of pulmonary involvement using clinical, radiological, functional, and genetic parameters in a well-characterized cohort receiving long-term ERT. The single-center study and relatively small sample size may limit generalizability, while the cross-sectional design precludes assessment of disease progression and the longitudinal effects of therapy. The absence of lung volume and DLCO measurements may have underestimated functional impairment, and the lack of serial HRCT limits assessment of radiological progression.

## Data Availability

The datasets generated during and/or analyzed during the current study are available from the corresponding author upon reasonable request.
